# Exploration of potential targets and mechanisms of Naringenin in treating autism spectrum disorder via network pharmacology and molecular docking

**DOI:** 10.1097/MD.0000000000031787

**Published:** 2022-11-18

**Authors:** Jialin Gai, Jinxiao Xing, Yangyang Wang, Junfang Lei, Chengdong Zhang, Jinfei Zhang, Jiqin Tang

**Affiliations:** a School of Rehabilitation Medicine, Shandong University of Traditional Chinese Medicine, Jinan, Shandong, China; b College of Rehabilitation Medicine, Weifang Medical University, Weifang, Shandong, China.

**Keywords:** autism, autism spectrum disorder, molecular docking, Naringenin, network pharmacology

## Abstract

Naringenin (NR) is a kind of flavonoid which plays a great role in the treatment of autism spectrum disorder (ASD). However, the underlying mechanism of NR in treating ASD still remains unclear. This study used network pharmacology and molecular docking to examine the potential targets and pharmacological mechanism of NR on ASD. Targets related to NR were screened from Traditional Chinese Medicine System Pharmacology Database and Analysis Platform (TCMSP), Encyclopedia of Traditional Chinese Medicine Database (ETCM), Traditional Chinese Medicine Integrated Database (TCMID), PharmaMapper database, and targets related to ASD were screened from Online Mendelian Inheritance In Man (OMIM), Disgenet, GeneCards, Therapeutic Target Database (TTD), Drugbank, and ETCM. Screened of the intersected gene targets. Then, we used the protein–protein interaction (PPI) networks to construct a PPI network and used Network Analyzer plug-in to perform topological analysis to screen out the core target. We used Metascape platform to perform gene ontology (GO) functional enrichment analysis and Kyoto Encyclopedia of Genes and Genomes (KEGG) pathway enrichment analysis, and used Chem draw, Pymol, AutoDock 1.5.6 software for molecular docking verification with core targets. A total of 149 targets of NR and 1594 potential targets of ASD were screened, and 43 intersected targets and 8 key targets were obtained and screened. A total of 176 GO items were obtained by GO enrichment analysis (*P* < .05), 153 entries on biological process (BP), 12 entries on BP and 11entries on cell composition (CC) were included. A total of 100 signaling pathways were obtained by KEGG pathway enrichment screening (*P* < .05).The pathways that are closely related to the pathogenesis of ASD are estrogen signaling, thyroid hormone signaling pathway, prolactin signaling pathway, and endocrine resistance pathway. Molecular docking results showed that NR had the best docking activity with the core target CASP3, and had good binding ability with AKT1, ESR1, ACTB and MAPK3. Taken together, our findings support that NR exerts therapeutic effects on ASD with multi-target, and multi-pathway characteristics, which provides a preliminary theoretical basis for clinical trials. The mechanism of anti-oxidative stress response, anti-apoptosis, regulation of cell growth and metabolism, anti-inflammatory, balance hormone levels may be important for the therapeutic effect.

## 1. Introduction

Autism Spectrum Disorder (ASD) is a complex chronic neurodevelopmental disease that occurs in childhood and is accompanied by persistent deficits such as various social impairments, behavioral impairments, language communication impairments, and repetitive stereotyped behaviors. These include anxiety, intellectual disability, motor abnormalities, epilepsy, attention disturbance, hyperactivity, sleep disturbance, immune system deregulation, and gastrointestinal problems.^[1,2]^ ASD has become increasingly common in recent years, with a reported global incidence rate of about 1% to 2%.^[3]^ In China, autistic children account for about 36.9% of mentally ill children, which brings a heavy economic burden to society and their families.^[4]^ Clinical treatment with western medicine consists primarily of drug combinations and behavior modification, educational intervention, speech training, and modern rehabilitation training. The target drugs for this disease are also in the exploratory stage, requiring a prolonged disease course to achieve curative effect while growing evidence suggests that traditional NR can be beneficial.^[5]^

In ancient Chinese medicine books, “dementia,” “late speech,” “fetal weakness,” “coma” and “depression syndrome” were assigned to the autism category. The Traditional Chinese Medicine (TCM) pathogenesis is based on deficiency and excess and is closely related to the brain, heart, kidney, and liver, involving qi deficiency, essence deficiency, blood deficiency, fire (heat), and phlegm-dampness, which can easily lead to brain disorders and insufficiency.^[6,7]^ Children with ASD often show typical heart and spleen deficiency symptoms and gastrointestinal problems such as food intolerance, food allergy, constipation or diarrhea, partial eclipse, and picky eating.^[8]^ TCM often involves a combination of syndrome differentiation and syndrome differentiation, which can effectively improve the core symptoms of children. NR is the main natural dihydroflavonol in various TCMs, such as tangerine peel. It is easily absorbed by the intestinal tract and has anti-inflammatory, anti-oxidative, lipid-regulating, and anti-apoptotic activities, can improve immunity, repair DNA damage and eliminate free radicals in the body, which can protect nerve and gastrointestinal function.^[9]^ Researchers such as Ranjana Bhandari established the pharmacokinetic and pharmacodynamic model to found that NR as an adjunct neurotherapeutic moiety in attenuating neuropsychopathology associated with ASD, such as sensorimotor dysfunction, hyperlocomotion, anxiety-like behaviors, social interactions, and repetitive behaviors.^[10]^ Likewise, it indicates anti-hyperlipidemic, anti-depressant, anti-proliferative effects and its effect on metabolic disorders and cognitive dysfunction which provides a pharmacological and experimental foothold for the prevention and treatment of ASD.^[11–14]^

**Figure 1.**
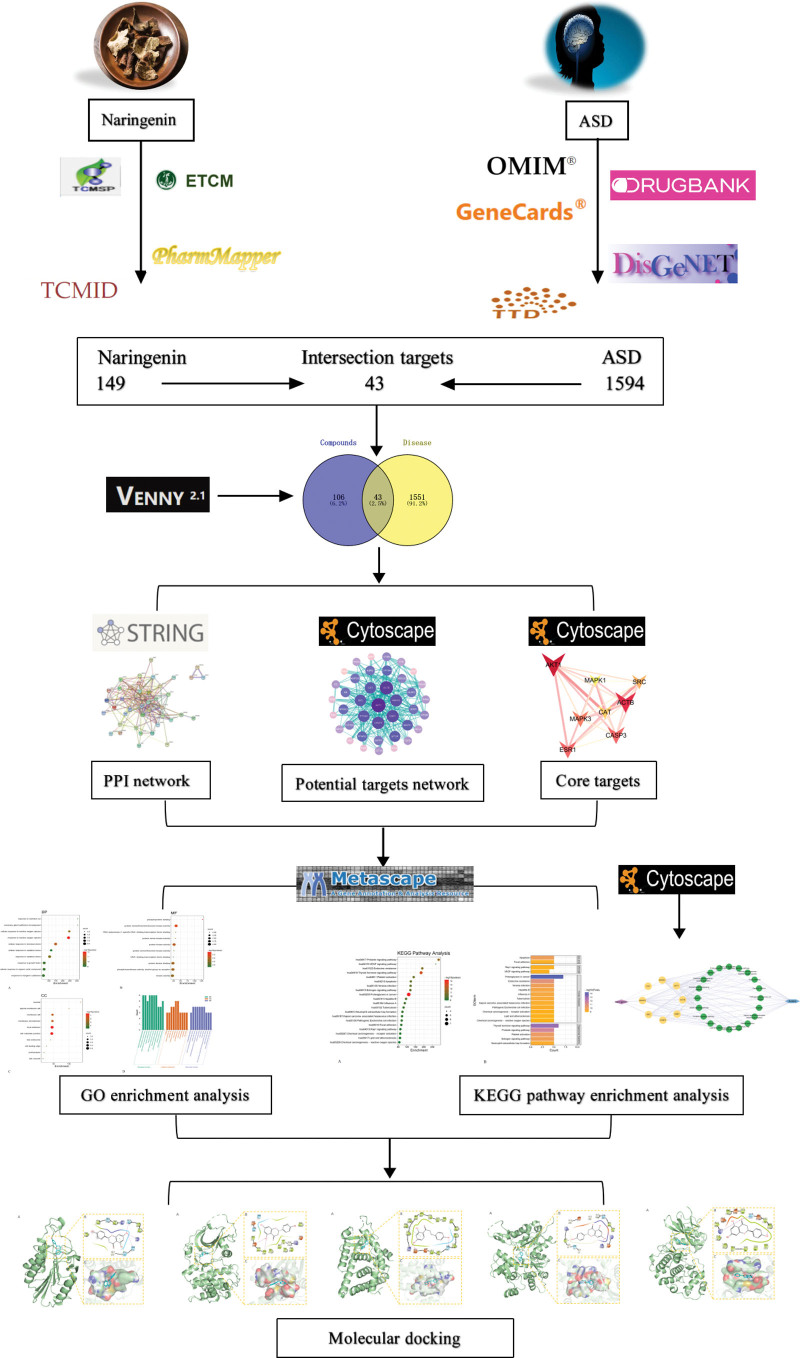
The flowchart of the present study. ASD = autism spectrum disorder, PPI = protein–protein interaction, GO = gene ontology, KEGG = Kyoto Encyclopedia of Genes and Genomes.

NR in treating ASD therapeutic potential has been reviewed,^[15]^ but the targets, pathway, and mechanism remain unclear. Hitherto, few specific studies have been conducted. As an emerging frontier science for TCM research, network pharmacology can integrate and systematize drugs, targets, and diseases and also embodies the overall concept and dialectical thinking of TCM concepts. Therefore, this study sought to explore the potential targets and molecular mechanisms of NR in the treatment of ASD from multiple perspectives, with network pharmacology and molecular docking technology, hoping to provide a scientific theoretical basis for the optimization of autism drug selection in the future and also lay a theoretical basis for subsequent experimental research (Fig. 1).

## 2. Materials and methods

### 2.1. Obtain NR structure and predict targets information

The TCMSP^[16]^ (http://tcmspw.com/tcmsp.php) was used to obtain the structure and target information of NR, and the PubChem database^[17]^ (https://pubchem.ncbi.nlm.nih.gov/) was used to obtain the Canonical simplified molecular-input line-entry system (SMILES) number of NR, which was imported into the Swiss Target Prediction database^[18]^ (http://www.swisstargetprediction.ch) for target prediction.

### 2.2. Screening intersected gene targets related to NR and ASD

#### 2.2.1. Screening of the target genes of NR.

The target genes of NR were screened from ETCM^[19]^ (http://www.tcmip.cn/ETCM/), TCMID^[20]^ (http://www.megabionet.org/tcmid/), and PharmaMapper^[21]^ (http://lilab.ecust.edu.cn/pharmmapper/) platform using “naringin” as the keyword, which were merged to remove duplicates, calibrated and compared through the Uniprot database^[22]^ (http://www.uniprot.org/). After unifying the ID name, we finally obtained the corresponding compound target genetic information.

#### 2.2.2. Screening of ASD-related genes.

By using “Autism Spectrum Disorder” as the keyword, we searched 6 disease databases of OMIM (http://omim.org/), Disgenet (https://www.disgenet.org/), GeneCards (http://www.genecard.org/), TTD (http://bidd.nus.edu.sg/group/cjttd), Drugbank (http://www.drugbank.ca), and ETCM to obtain ASD-related genes,^[23–27]^ and the target names were standardized through the Uniprot database. Then, the target names were combined and deduplicated to obtain ASD-related genes.

#### 2.2.3. Screening of the intersected gene targets.

The NR action targets and disease targets retrieved above were imported into Venny 2.1.0 (https://bioinfogp.cnb.csic.es/tools/venny) to obtain the intersected genes, and a Venn plot was created to visualize the potential targets of NR in the treatment of ASD.

### 2.3. Construction of protein–protein interactions (PPI) network

The construction of PPI is crucial for understanding the indication to what extent 2 proteins interact with each other.^[28]^ We imported the potential targets obtained above into the STRING^[29]^ (https://string-db.org) platform. “Organism” was set to “Homo Sapiens,” free nodes were hidden, and PPI network was constructed to derive node information to prepare for the next step, which involved generating a network graph to explore the interaction between NR-related targets and ASD targets.

### 2.4. Screening of core targets

In order to obtain core target genes, we used the tool Network Analyzer in Cytoscape 3.9.0^[30]^ to analyze the network topology parameters. The node size and color depth reflected the degree value. And the key targets were filtered through the plug-in centiscape2.2^[31]^ by calculating the mean values of betweenness, closeness, and degree. The targets above the 3 thresholds of betweenness, closeness, and degree were selected as “key targets,” which could be considered as the core target genes of NR in the treatment of ASD.

### 2.5. GO and KEGG pathway enrichment analysis

GO biological function enrichment analysis and KEGG metabolic pathway enrichment analysis were performed on the screened key targets through the Metascape platform^[32]^(https://metascape.org/). “Homo Sapiens” (human source) was selected for the species background of Metascape; the *P*-value was set to <0.01, the Min Overlap value was 3, and the Min Enrichment value was 1.5. GO annotation was conducted on 3 aspects: BP, CC, and molecular function (MF). The microscopic letters (http://www.bioinformatics.com.cn) generated the bubble plots and histogram, and the Cytoscape 3.9.0 platform was used to draw the “component-target-disease-pathway” network diagram.

### 2.6. Molecular docking

#### 2.6.1. Preparation of target proteins and small molecule structures.

We conducted a molecular docking analysis of the key targets screened in 1.4 with the NR component. The structure of the NR compound was obtained from the PubChem database. The structure optimization was performed by Chem3D 17.0 software; the energy of the downloaded compound was minimized and converted into mol2 format. Small molecule compounds were imported into AutoDock Tools-1.5.6 software and saved as Protein Data Bank (PDB) files after adding atomic charges, assigning atomic types, and making all flexible bonds rotatable by default.

The crystal structure of the target protein was obtained from the Uniprot protein database. After using Pymol 2.1 software to delete irrelevant small molecules in the protein molecule, the protein molecule was imported into AutoDock Tools-1.5.6 software to delete water molecules, add hydrogen atoms, set the atom type, and finally saved as PDB files.

#### 2.6.2. Molecular docking process.

The treated compound was used as a small molecule ligand, and 8 protein targets were used as receptors. The center position and length, width, and height of the Grid Box were set to 50 × 50 × 50 according to interactions between the small molecule and the target. Finally, molecular docking was performed by AutoDock Tool software to verify their binding efficiency, and a Lamarckian genetic algorithm was used for molecular docking calculation.

## 3. Result

### 3.1. Pharmacological and molecular structure information of NR

**Figure 2.**
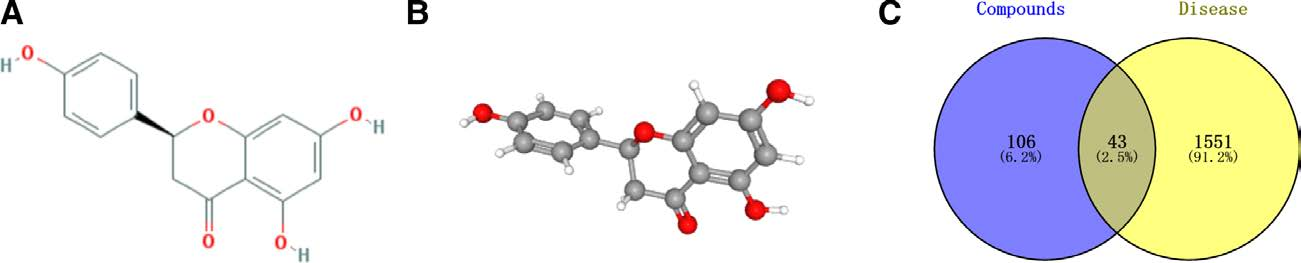
(A) The 2D structure of NR, (B) the 3D structure of NR, and (C) NR-ASD Intersection Target VENN Map. Compounds = NR (blue), Disease = ASD (yellow). A total of 43 gene products were identified as common. ASD = autism spectrum disorder, NR = Naringenin.

Through the TCMSP and the PubChem database, we obtained the structure of NR. The 2D and 3D structures of NR are shown in Figure 2A and B. Its molecular ID is MOL004328, molecular formula C_15_H_12_O_5_, molecular weight 272.27, the ratio of lipid water partition coefficient (AlogP) 2.30, OB 59.29, blood-brain barrier (BBB) −0.37, and drug-likeness (DL) 0.21, drug half-life (HL) 16.98.

### 3.2. Screening of the intersected gene targets related to NR and ASD

Thirty-six potential targets of NR were obtained from the TCMSP database, 29 potential targets were obtained from the Swiss Target Prediction database, and 44, 48, 65 potential targets of NR were obtained from ETCM, TCMID, and PharmaMapper, respectively. After the targets obtained in the above databases were merged and deduplicated, 149 NR-related targets were obtained.

Ninety-one, 1113, 733, and 68 ASD-related targets were obtained from the OMIM, Disgenet, GeneCard, and Drugbank databases, respectively, and combined with the 2 databases of TTD and ETCM to supplement related targets. A total of 1594 ASD disease targets were obtained after merging and deleting duplicate targets.

The screened NR and ASD-related targets were mapped, and the intersection of the 2 was obtained, yielding 43 targets, and a “drug-disease” Venn plot was generated in Figure 2C. The potential targets of NR in the treatment of ASD are shown in Table 1.

**Table 1:** Potential target genes of NR in the treatment of autism.

Number	Gene	Number	Gene
1	ESR1	23	SHBG
2	PTGS2	24	NLRP3
3	PIK3CG	25	NFE2L2
4	AKT1	26	MTOR
5	BCL2	27	MAOB
6	MAPK3	28	ABCG2
7	MAPK1	29	ADORA2A
8	CASP3	30	AR
9	SOD1	31	TTR
10	CAT	32	CTSB
11	PPARG	33	PDE4D
12	APOB	34	F2
13	CYP19A1	35	PPARD
14	GSTP1	36	FGFR1
15	UGT1A1	37	PAH
16	ADIPOQ	38	SRC
17	ABAT	39	HK1
18	SOAT1	40	PDE4B
19	ACTB	41	AMY1A
20	CYP1B1	42	AMY1B
21	ESR2	43	AMY1C
22	NR1I2		

NR = Naringenin.

### 3.3. Construction of PPI network of NR against ASD

**Figure 3.**
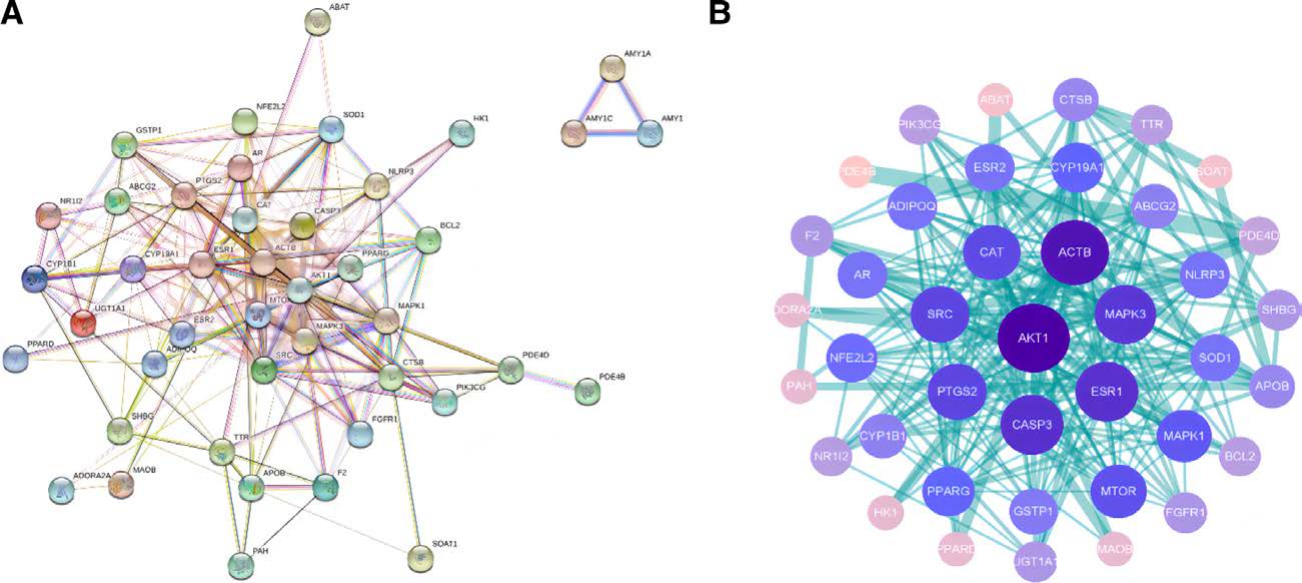
(A) PPI network, (B) PPI network diagram of potential target degree value. The node size is proportional to the degree. PPI = protein–protein interaction.

The protein interaction PPI information obtained from the STRING database was imported into Cytoscape 3.9.0 software as a “TSV” file to generate a PPI network map of potential targets involving 40 nodes and 234 edges (Fig. 3). The node size and color depth in the figure represent the node degree value. A larger node and darker color corresponded to a larger node degree value. The thicker and darker the edge, the higher the interconnectivity and the greater its role in the network. The results showed that the average node degree of the network was 11.7, and the clustering coefficient was 0.593.

### 3.4. The screening results of core targets network

**Figure 4.**
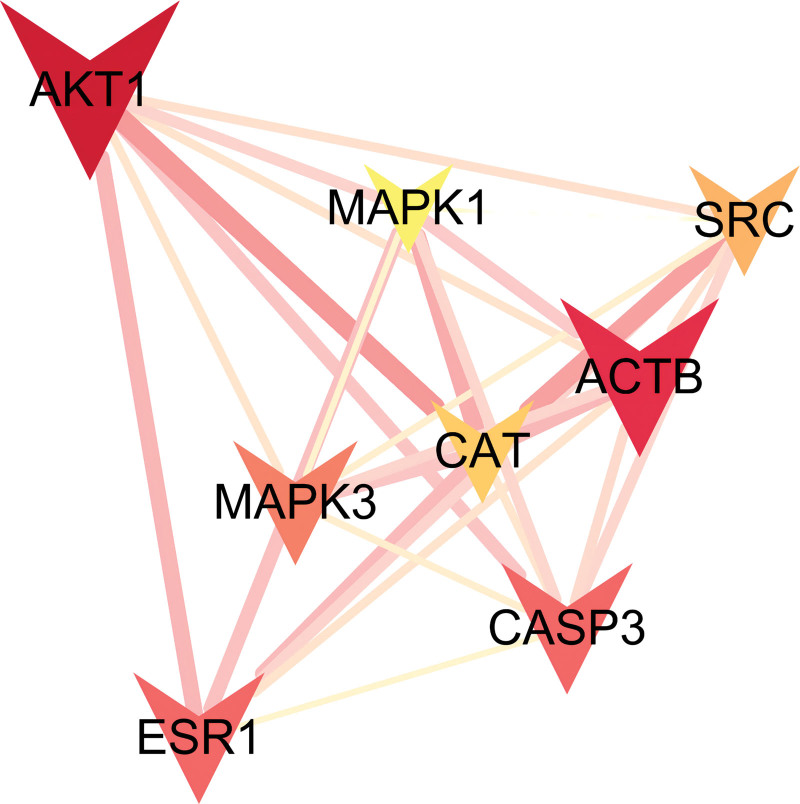
Core targets PPI network diagram, the thicker the edge, the closer the relationship among proteins. PPI = protein–protein interaction.

In Cytoscape3.9.0 software, the plug-in centiscape2.2 was used to calculate betweenness, closeness, and degree values. The betweenness, closeness, and degree values were 0.014236605, 33.5, and 11.7. Targets with values above the 3 thresholds were set as key targets (Fig. 4). The details are shown in Table 2. Targets that met the above requirements were AKT1, ACTB, ESR1, CASP3, MAPK3, SRC, CAT, and MAPK1, suggesting they are closely related to other target proteins. Based on this, we hypothesized that they might be involved in the pharmacological effects of NR in the treatment of ASD.

**Table 2:** Core target information.

Number	Core target	Betweenness unDir	Closeness unDir	Degree unDir
1	CAT	98.60251676	0.016666667	30
2	MAPK3	62.83480866	0.017241379	27
3	SRC	59.50302501	0.016949153	23
4	CASP3	76.96309488	0.01754386	23
5	ESR1	64.03900537	0.017241379	22
6	ACTB	141.6436652	0.019230769	20
7	AKT1	268.8019609	0.020833333	19
8	MAPK1	38.27931872	0.016129032	17

### 3.5. KEGG pathway analysis and GO enrichment analysis

**Figure 5.**
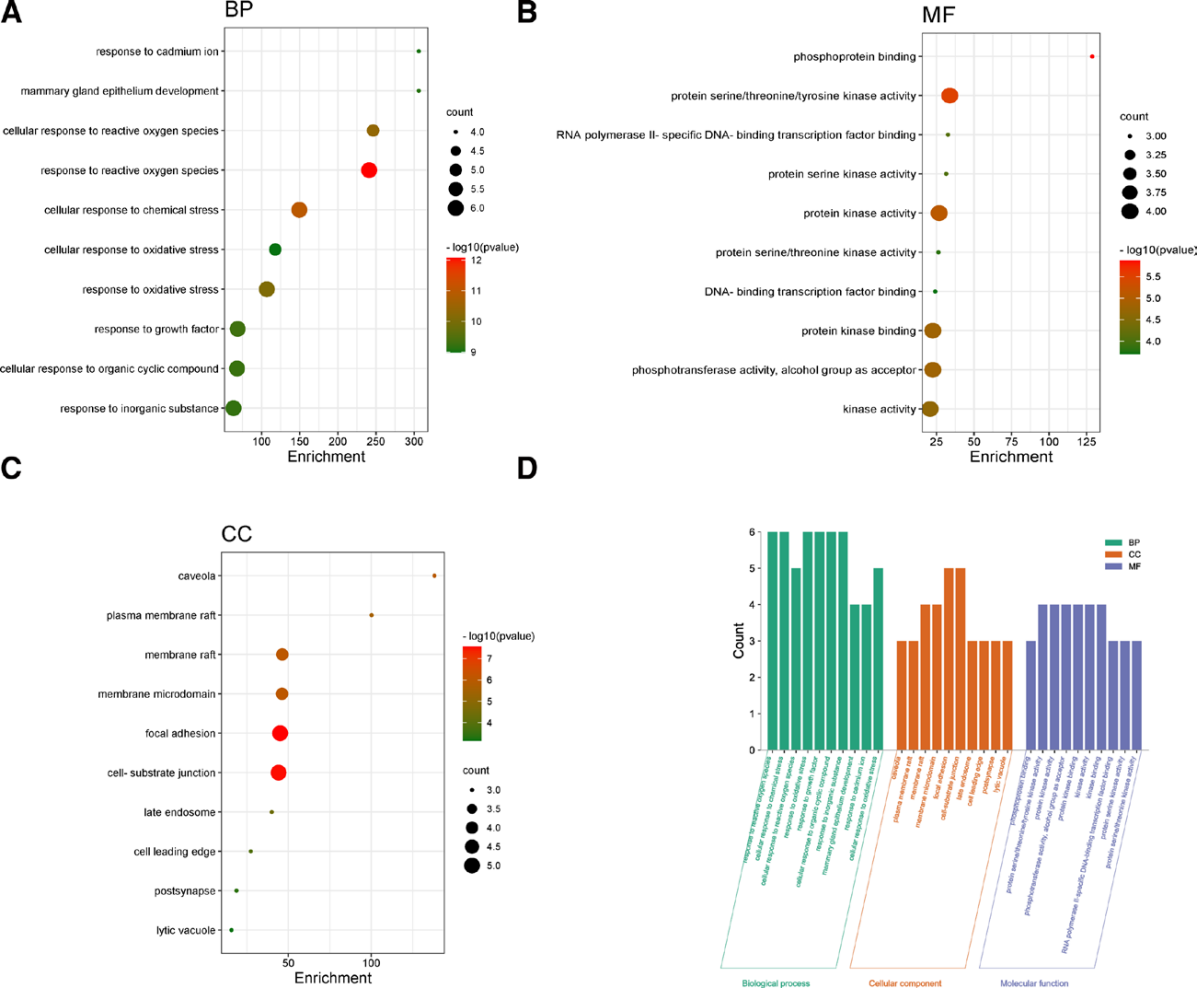
(A) GO function enrichment analysis bubble diagram. The circle size represents the number of enriched genes; color represents the significance of enrichment. BP = biological process, (B) MF = molecular function, (C) CC = cell component, (D) Histogram of GO enrichment analysis of NR in the treatment of ASD. ASD = autism spectrum disorder, GO = gene ontology.

The screened core targets were imported into the Metascape database for GO gene function annotation and KEGG pathway enrichment analysis. The top 10 most significant GO terms according to the count are shown in Figure 5A–C, and display the bubble chart according to the *P* value, as shown in Figure 5D. During GO annotation, a total of 176 GO terms were obtained for BP (n = 153), CC (n = 11), and MF (n = 12). Significantly enriched biological processes consisted of response to reactive oxygen species (ROS), chemical stress, oxidative stress and growth factors, and organic cyclic compounds. Significantly enriched CC GO terms consisted of focal adhesion, cell-substrate junction, membrane raft, membrane microdomain, caveola, plasma membrane raft, etc. Finally, the significantly enriched MF GO terms included phosphoprotein binding, protein serine/threonine/tyrosine kinase activity, RNA polymerase II-specific DNA-binding transcription factor binding, protein kinase binding, kinase activity, etc.

**Figure 6.**
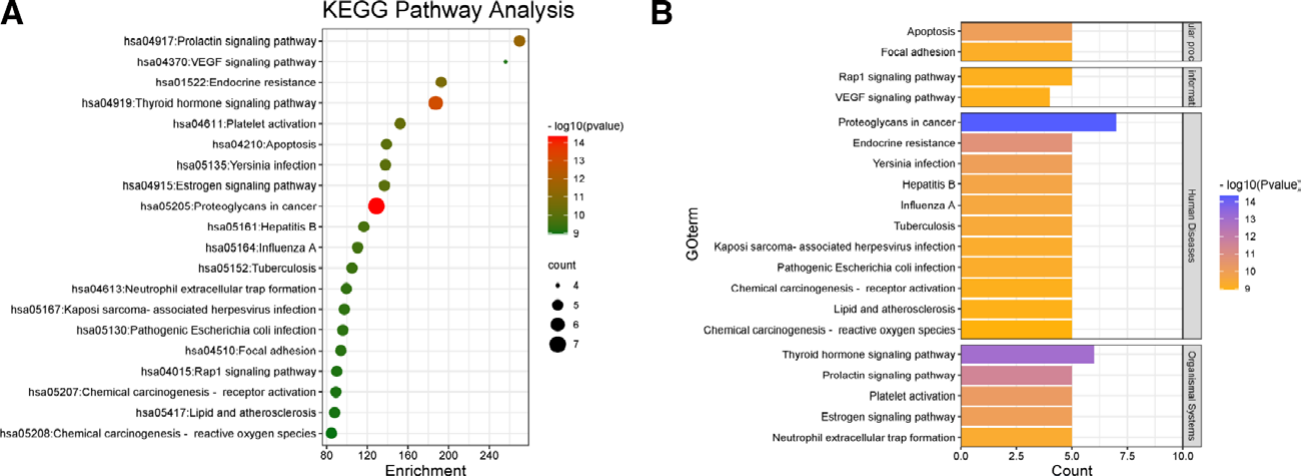
(A) Bubble plot of KEGG pathway enrichment analysis of NR – ASD, (B) KEGG pathway classification plot of NR-ASD. The 4 modules from top to bottom are cellular process, environmental information processing, Human Diseases, and Organismal Systems. ASD = autism spectrum disorder, KEGG = Kyoto Encyclopedia of Genes and Genomes, NR = Naringenin.

The KEGG pathway enrichment analysis revealed 100 enriched metabolic pathways (*P* < 0.01), the top 20 representative pathways were selected for bubble diagram according to the *P*-value, as shown in Figure 6A, and the pathways were classified according to KEGG pathway classification, as shown in Figure 6B,and the specific information is provided in Table 3. KEGG network includes the first 20 pathways, of which MAPK1, MAPK3, AKT1, SRC, ACTB and other genes appear the most. It involves prolactin signaling pathway, estrogen signaling, proteoglycans in cancer, thyroid hormone signaling pathway, endocrine resistance, apoptosis, platelet activation, VEGF signaling pathway, Yersinia infection, etc.

**Table 3:** KEGG metabolic pathway enrichment analysis of NR-autism spectrum disorder.

Pathway	Enrichment	*P*-value	Count	Gene
Proteoglycans in cancer	128.9493902	4.75E–15	7	ACTB AKT1 CASP3 ESR1 MAPK1 MAPK3 SRC
Thyroid hormone signaling pathway	187.2582645	1.01E–13	6	ACTB AKT1 ESR1 MAPK1 MAPK3 SRC
Prolactin signaling pathway	269.7410714	3.22E–12	5	AKT1 ESR1 MAPK1 MAPK3 SRC
Endocrine resistance	192.6721939	1.80E–11	5	AKT1 ESR1 MAPK1 MAPK3 SRC
Platelet activation	152.2731855	5.95E–11	5	ACTB AKT1 MAPK1 MAPK3 SRC
Apoptosis	138.8373162	9.51E–11	5	ACTB AKT1 CASP3 MAPK1 MAPK3
Yersinia infection	137.8239051	9.87E–11	5	ACTB AKT1 MAPK1 MAPK3 SRC
Estrogen signaling pathway	136.8251812	1.02E–10	5	AKT1 ESR1 MAPK1 MAPK3 SRC
Hepatitis B	116.554784	2.30E–10	5	AKT1 CASP3 MAPK1 MAPK3 SRC
Influenza A	110.4203216	3.03E–10	5	ACTB AKT1 CASP3 MAPK1 MAPK3
Tuberculosis	104.8993056	3.92E–10	5	AKT1 CASP3 MAPK1 MAPK3 SRC
Neutrophil extracellular trap formation	99.37828947	5.15E–10	5	ACTB AKT1 MAPK1 MAPK3 SRC
Kaposi sarcoma-associated herpesvirus infection	97.32925258	5.72E–10	5	AKT1 CASP3 MAPK1 MAPK3 SRC
Pathogenic Escherichia coli infection	95.84708122	6.18E–10	5	ACTB CASP3 MAPK1 MAPK3 SRC
Focal adhesion	93.93967662	6.83E–10	5	ACTB AKT1 MAPK1 MAPK3 SRC
Rap1 signaling pathway	89.91369048	8.52E–10	5	ACTB AKT1 MAPK1 MAPK3 SRC
Chemical carcinogenesis - receptor activation	89.06544811	8.93E–10	5	AKT1 ESR1 MAPK1 MAPK3 SRC
VEGF signaling pathway	256.0254237	9.13E–10	4	AKT1 MAPK1 MAPK3 SRC
Lipid and atherosclerosis	87.82267442	9.59E–10	5	AKT1 CASP3 MAPK1 MAPK3 SRC

KEGG = Kyoto Encyclopedia of Genes and Genomes, NR = Naringenin.

### 3.6. “NR-Target-ASD-pathway” PPI network diagram

**Figure 7.**
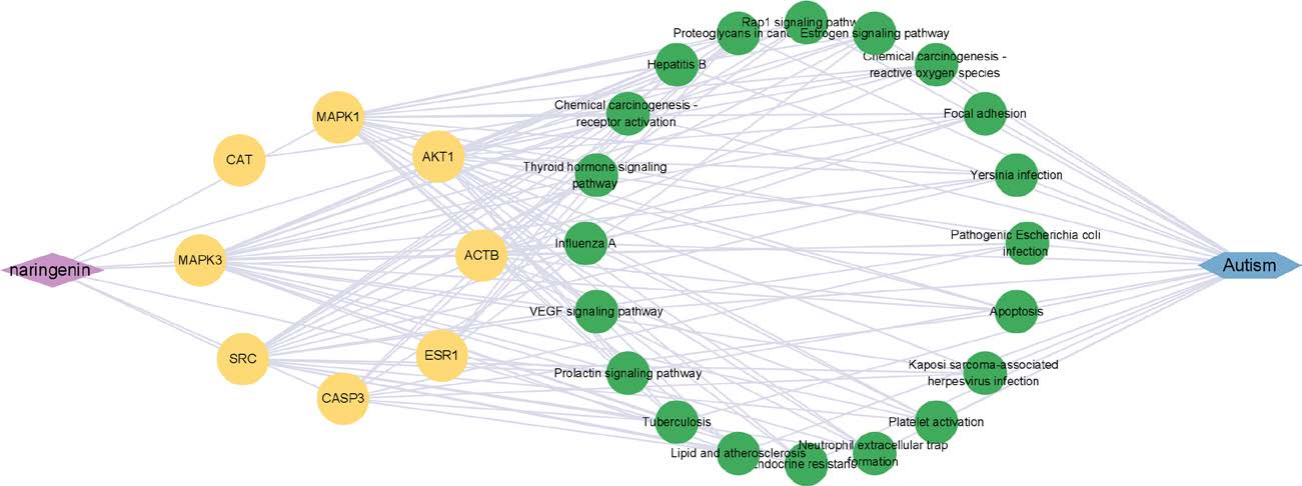
Target-pathway PPI network of NR in the treatment of ASD. ASD = autism spectrum disorder, NR = Naringenin, PPI = protein-protein interaction.

The “NR-Target-ASD-Pathway” PPI network diagram is shown in Figure 7. The purple diamond node on the left represents the NR compound, the yellow circle icon in the middle represents the key target, the green icon in the middle represents the representative top 20 signaling pathways where the target is located, and the blue rectangle icon on the right represents autism. After further topological analysis by Network Analyzer, we found that NR may play a co-regulatory role on ASD mediated by AKT1, MAPK1, MAPK3 and involve the proteoglycan pathway in cancer, thyroid hormone signaling pathway, ROS pathway, and other pathways.

### 3.7. The results of molecular docking

To further explore the interaction between NR and key targets, the NR compound and key targets AKT1, ACTB, CAT, ESR1, MAPK1, MAPK3, CASP3, and SRC were molecularly docked. The results of the final molecular docking study are shown in Table 4. It is well-established that binding energy can predict the binding activity between 2 parties. Indeed, the lower the binding energy, the tighter the compound binds to the target protein, and the more stable their conformation is. Binding energy <−5 kcal/mol is associated with good binding ability, and binding energy <−7 kcal/mol suggests strong activity.^[33]^ As seen in Table 4, the binding energies of all targets and compounds were <−5 kcal/mol, indicating that NR has a good binding effect with these target proteins and has a high degree of matching. The binding energies of the 5 target proteins CASP3, AKT1, ESR1, ACTB, and MAPK3 were all <−7kcal/mol and exhibited the strongest binding ability to the compound, suggesting that NR plays an essential role in ASD through these targets.

**Table 4:** The docking results for each target with compound (kcal/mol).

Compounds	Structure	Target ID	PDB ID	Docking Score/kcal/mol	Combination Type
NR	C_15_H_12_O_5_	CASP3	1NMS	–7.59	Hydrogen bonds, Hydrophobic interactive, π-stacking
		AKT1	4GV1	–7.54	
		ESR1	3ERT	–7.48	
		ACTB	6NBW	–7.18	
		MAPK3	5W62	–7.16	
		MAPK1	6SLG	–6.87	
		SRC	6ATE	–6.31	
		CAT	1DGF	–5.63	

NR = Naringenin, PDB = Protein Data Bank.

**Figure 8.**
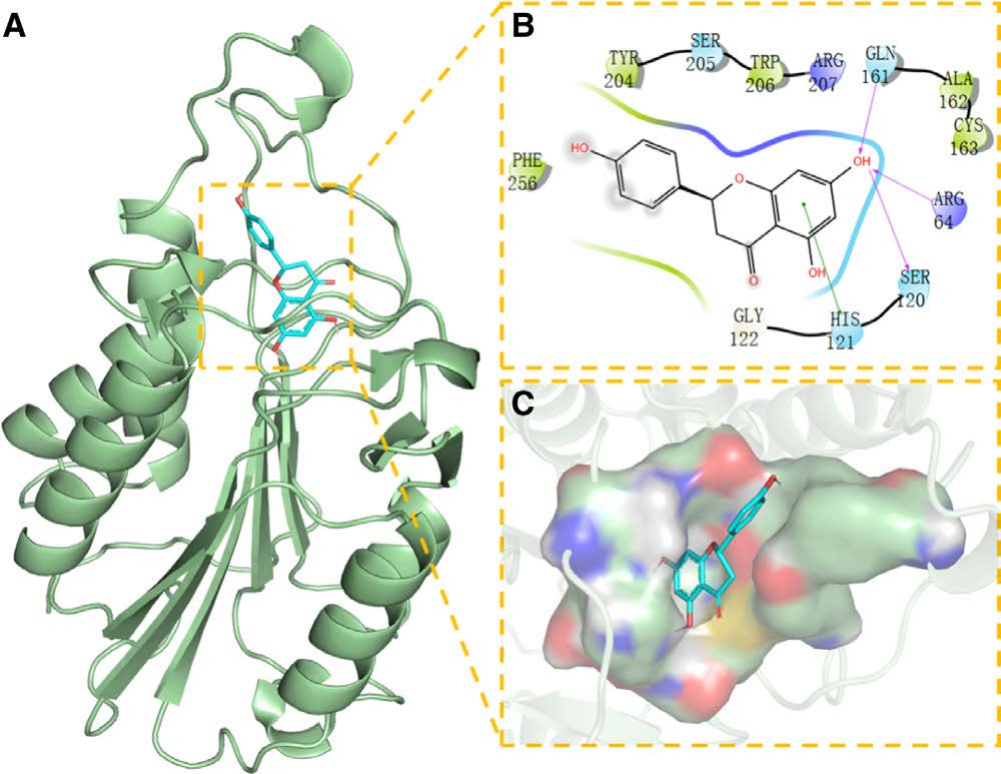
Docking of CASP3 with NR. (A) 3D structure of complex, (B) the hydrogen bond donor receptor network of complex, and (C) 2D binding mode of complex. NR = Naringenin.

**Figure 9.**
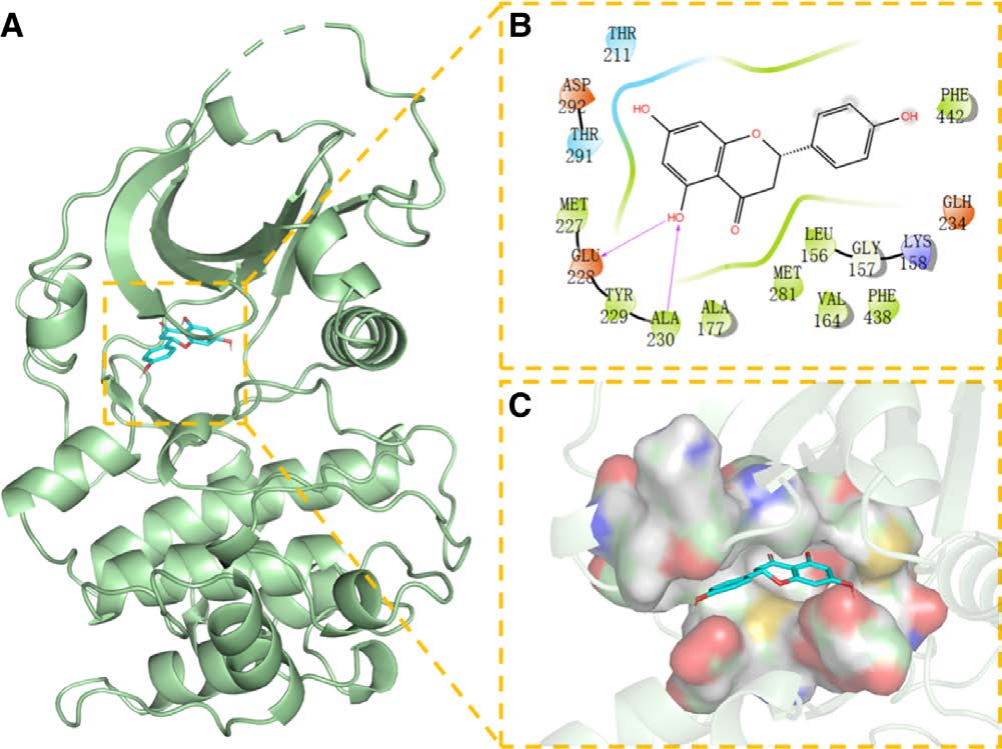
Docking of AKT1 with NR. (A) 3D structure of complex, (B) the hydrogen bond donor receptor network of complex, and (C) 2D binding mode of complex. NR = Naringenin.

**Figure 10.**
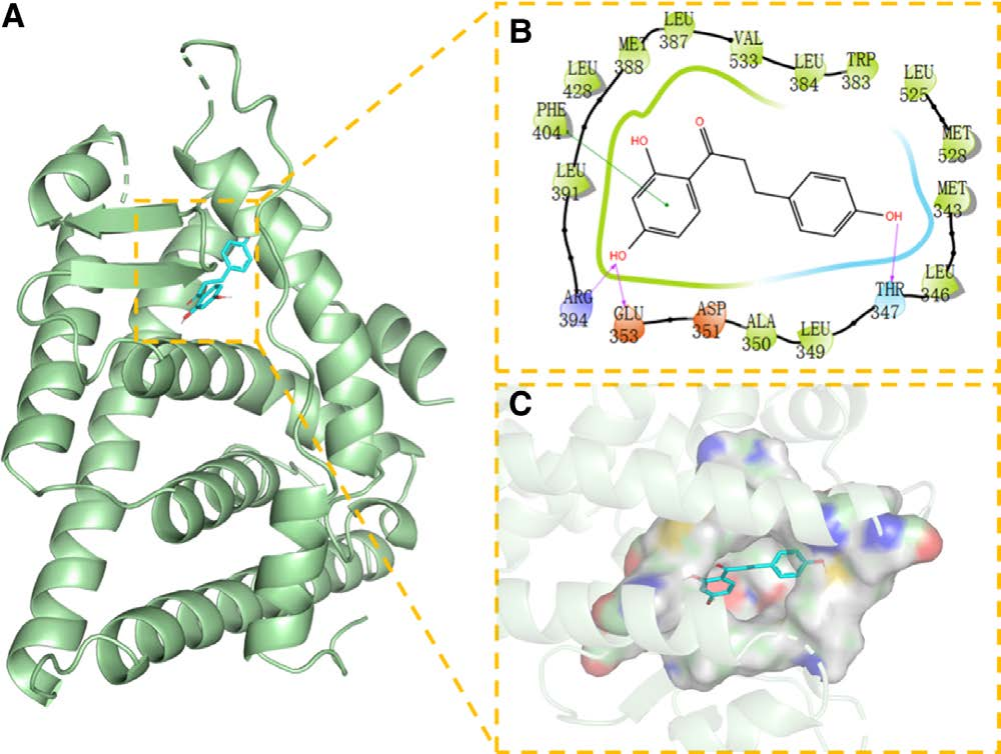
Docking of ESR1 with NR. (A) 3D structure of complex, (B) the hydrogen bond donor receptor network of complex, and (C) 2D binding mode of complex. NR = Naringenin.

**Figure 11.**
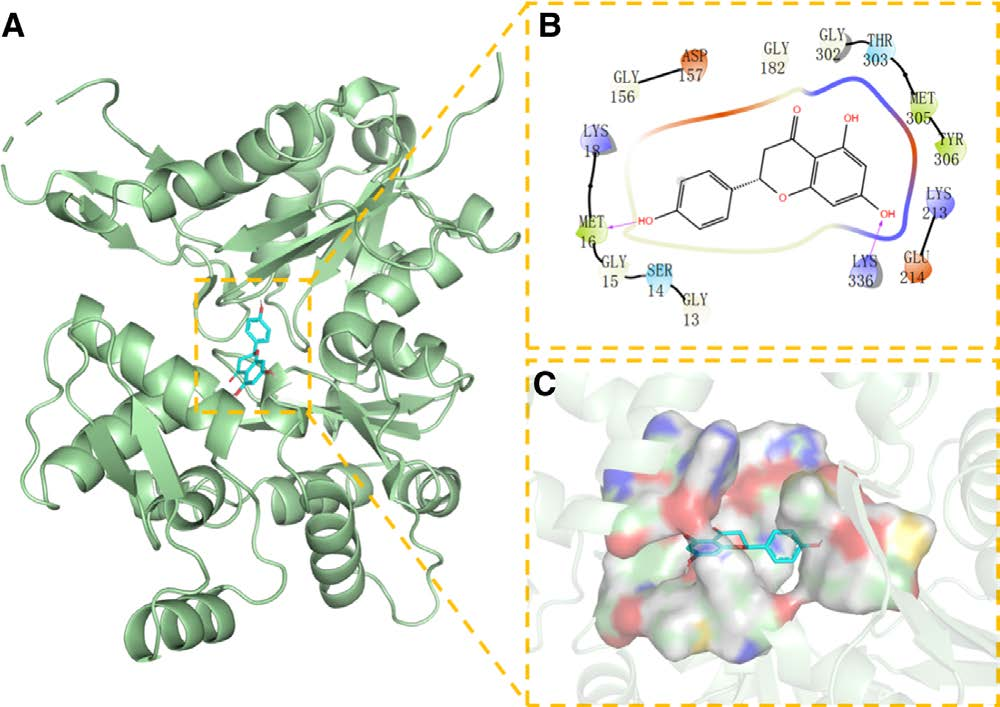
Docking of ACTB with NR. (A) 3D structure of complex, (B) the hydrogen bond donor receptor network of complex, and (C) 2D binding mode of complex. NR = Naringenin.

**Figure 12.**
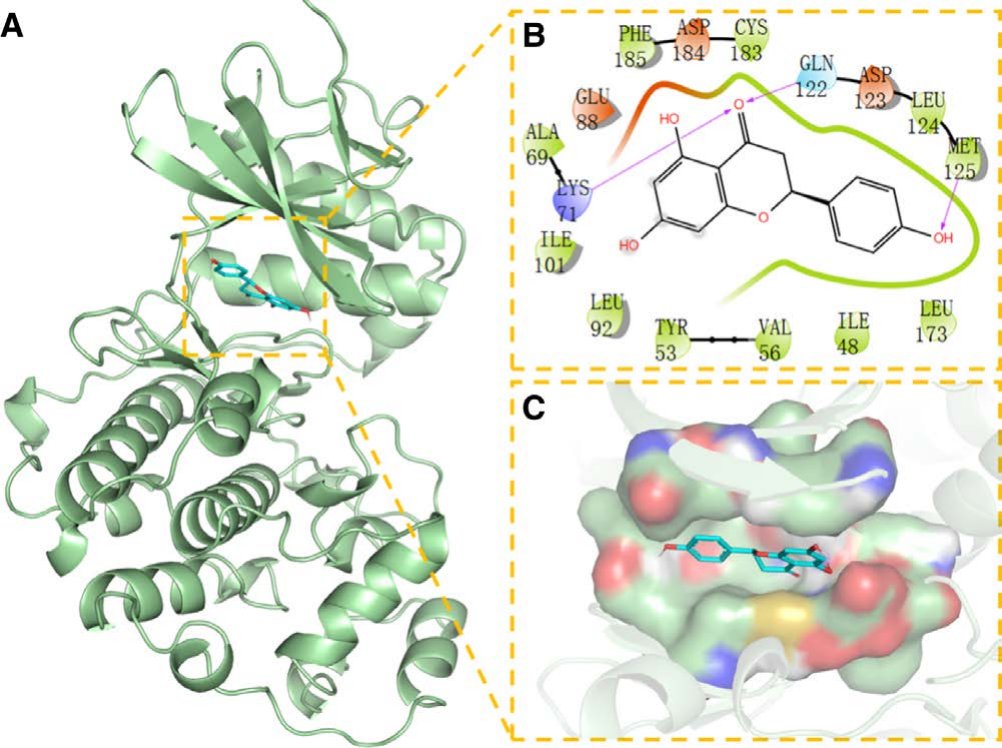
Docking of MAPK3 with NR. (A) 3D structure of complex, (B) the hydrogen bond donor receptor network of complex, and (C) 2D binding mode of complex. NR = Naringenin.

The complex formed by the compound and the protein after docking was visualized with Pymol2.1 software, and the binding mode of the compound and the protein was obtained. According to the binding mode, we can clearly see the amino acid residues that the compound binds to the protein pocket. Molecular docking details of NR with each protein are shown in Figures 8–12.Then we selected the top 3 target proteins with the lowest binding energies for analysis.

We found that NR has the best match with the active site of CASP3 protein; its phenolic hydroxyl group could form multiple hydrogen bonds with SER-120, GLLN-161, ARG-64, yielding a strong effect on protein stability. In addition, the benzene ring of this compound can form strong π-π conjugated interactions with the benzene ring of TYR-204, playing an essential role in the stabilization of small molecules. The amino acid residues that interact with the active site of NR and AKT1 protein are THR-291, MET-227, GLU-228, TYR-229, ALA-230, ALA-177, LEU-156, VAL-164, PHE-438, and PHE-44. NR has strong hydrophobicity and can form strong hydrophobic interactions with amino acids in the active site (MET-227, TYR-229, ALA-230, ALA-177, LEU-156, VAL-164, PHE-438, and PHE-442), playing an essential role in stabilizing small molecules in the protein cavity. This compound can also form hydrogen bonds with GLU-228 and ALA-230 amino acids, which contribute to the small molecules in the anchor protein cavity. Moreover, we found that NR and the ESR1 protein site can form hydrogen bonds, hydrophobic interactions, van der Waals forces, and other interactions; for example, NR can interact with LEU-387, PHE-404, LEU-391, ALA-350, VAL-533, MET -343, LEU-346, and other amino acids to form strong hydrophobic interactions, and can form strong hydrogen bond interactions with LEU-387, ARG-394, GLU-353.

In addition, NR can interact with several other targets in various ways, effectively promoting the formation of stable complexes between proteins and small molecules and having strong associations with the targets.

## 4. Discussion

In recent years, there has been a burgeoning interest in exploring the underlying molecular and cellular mechanisms of ASD. Ample evidence suggests that its pathogenesis includes genetic, environmental, and immune factors. High genetic variability involving disruption of messenger ribonucleic acid (mRNA) transcription or protein function, deletion of gene exons, abnormal gene methylation, single nucleotide polymorphism, and copy number variation represents a significant risk factor for disease.^[34]^ Other risk factors include increasing parental age, influenza vaccine, drug infection or exposure to valproic acid and folic acid during pregnancy, maternal obesity, maternal smoking, preterm birth, low birth weight, and altered gut microbiota.^[35]^ The current treatment approach for ASD involves modulating neurotrophic factor levels, regulating gene expression, restoring cell synapse levels, restoring protein homeostasis, and stimulating oxidative stress responses.^[36]^

NR, chemically known as 5,7-dihydroxy-2-(4-hydroxyphenyl)chroman-4-one, is a disaccharide derivative at position 7 via a glycosidic linkage. It is a natural flavonoid glycoside with various pharmacological activities including high antioxidant, anti-inflammatory, anti-apoptotic and neuroprotective properties that can be found in a diversity of citrus fruits, vegetables, nuts and Chinese medicine.^[37]^ Flavonoid compounds show considerable potential and advantages for cancer prevention, cardiovascular diseases, and diabetes mellitus, mostly postulated to be due to their antioxidant effects.^[38]^ NR as a flavanone flavonoid that can have a protective effect on the gastrointestinal tract and effectively improve neurological diseases.^[39]^ Oral bioavailability (OB) is an important index to evaluate the effectiveness of drugs entering human circulation and the OB value of >30 is often used as a standard for compound screening.^[40]^ Our study found that the OB value of NR was 59.29, indicating that NR may be easily absorbed into the blood after oral administration, and it thus can conveniently exert biological activity. Some studies showed NR could improve spatial learning and memory in aging mice by inhibiting Aβ levels and neuroinflammation, phosphorylation, and oxidative stress.^[41]^ It can also downregulate anti-apoptotic genes to improve cognitive dysfunction, dyskinesia, depression, and anxiety-like behavior in hypoxic mice_._^[42]^

In our study, a total of 43 common targets of NR in the treatment of ASD were obtained through TCMSP, OMIM, and other related databases, and 8 key targets were obtained after screening. Topological analysis and molecular docking results showed that CASP3 had the closest binding ability to NR, and other targets such as AKT1, ESR1,ACTB, MAPK1, MAPK3, suggesting that their binding ability was also good. CASP3 (Caspase 3) is a group of proteases that play a key executive role during apoptosis, capable of eliminating redundant and nonfunctional synapses and removing extra cells during early childhood development, and play an important role in embryonic neuronal development.^[43]^ Experiments have shown that Casp3-deficient mice exhibit abnormal motor behaviors such as impaired social interaction, limited interest, and repetitive stereotypes associated with endoplasmic reticulum stress. Ample evidence substantiates that children with ASD have significantly higher levels of CASP3, which can lead to apoptosis in autistic brain tissue. NR can inhibit CASP3 elevations and alleviate endoplasmic reticulum stress and oxidative stress-induced apoptosis in ASD.^[44–46]^ Moreover, AKT1 (AKT serine/threonine kinase 1) reportedly regulates cell proliferation, metastasis, and apoptosis. Abnormalities in the PI3K/AKT/mTOR pathway have been associated with abnormal synaptic protein synthesis and the development of ASD. Current evidence suggests that NR can control cell apoptosis and maintain cell activity by regulating AKT1 target proteins.^[47]^ At the same time, NR can significantly inhibit AKT1 phosphorylation, promote gene transcription and protein synthesis, and activate Akt-mTOR signaling to inhibit apoptosis in autism and alleviate abnormal social behavior in mice.^[48,49]^

ESR1, a transcription factor of the nuclear receptor family, can mediate estrogen ligands, promote growth and cell survival, and is associated with social behavior and emotional regulation in children with ASD.^[50]^ Interestingly, women with abnormal antenatal testosterone and sex steroid precursor exposure have higher rates of autistic traits. Abnormal chemical stimuli into cells send signals at the membrane and nuclear levels through the Estrogen receptor, stimulate kinases and phosphatases in the body, change the phosphorylation state of key protein kinases, and reduce estrogen biosynthesis.^[51,52]^ Experiments have demonstrated that the ERɑ/β-mediated estrogen system of NR regulates oxytocin secretion in rodent hypothalamic neurons during rodent central nervous system development and can effectively activate ER-α inhibitory activity, reduce cellular stress through the inhibition of ESR1-mRNA expression or the downregulation of estrogen protein involving the corresponding signaling pathway.^[53]^ Moreover, estrogen-induced activation of the estrogen results in a reduction in the levels of nuclear DNA-binding activity of NF-kB, which in turn regulates the expression of inflammatory genes.^[54]^ Therefore, anti-inflammatory action as a potential mechanism in mediating the neuroprotective effects of estrogen.

GO and KEGG enrichment analysis enabled further analysis of their gene functions. The results showed that the therapeutic effect of NR in the treatment of ASD involves various pathways such as estrogen signaling, thyroid hormone signaling pathway, prolactin signaling pathway, endocrine resistance pathway, platelet activation pathway, and apoptosis pathway while involving different biological processes such as ROS metabolism, chemical stress, oxidative stress, cell growth, and apoptosis.

ROS are widely acknowledged as a major cause of cellular damage, premature cellular aging, and neurological diseases. ROS have been associated with increased activity of mitogen-activated protein kinases and play essential roles in cell growth, differentiation, development, cell cycle, survival, and cell death by activating the redox reaction of tyrosine kinases while affecting the activity of critical growth and metabolism-related transcription factors, and is sensitive to redox changes.^[55,56]^ Environmental and genetic risk factors may exacerbate ASD patients’ vulnerability to oxidative stress, impair antioxidant defense mechanisms, and lead to cell membrane damage, altered membrane fluidity, and permeability.^[57]^ For example, when a child is exposed to toxic air or environmental pollution in the mother’s body, an excess of free radicals is produced in the child’s body, leading to increased inflammation, toxicity, and oxidative stress. On the other hand, patients with autism exhibit blood-brain barrier leakage and are relatively sensitive to oxidative damage, leading to the manifestations of neuropsychiatric disorders, such as depression, cognitive dysfunction, psychosis, anxiety, etc.^[58]^ Recent studies found that NR is a flavonoid that has an inhibitory effect on signal transduction enzymes and controls cell growth by regulating proteins such as protein tyrosine and protein kinases.^[14]^ Moreover, NR possesses antioxidant properties, which can regulate the levels of ROS, inhibit oxidative stress and neuroinflammation, thereby improving cognition levels.

Interestingly, thyroid hormone signal transduction pathways have been shown to play important roles in metabolic processes such as central nervous system development and anti-apoptosis during early development.^[59]^ NR can reduce hormone levels through the thyroid hormone signaling pathway to restore normal cell growth and metabolism in children with ASD.^[60]^ In this regard, mothers who drink heavily or take antipsychotics for a long time during pregnancy can cause hormone imbalance, reduce oxytocin secretion, and have a higher chance of developing ASD in the baby. Overwhelming evidence substantiates that plasma oxytocin levels in autistic patients are lower than usual, and low levels of oxytocin can affect children’s social recognition behavior.^[61–63]^ It has also been reported that children with ASD have hypoplasia of the nervous system, accompanied by inflammation, oxidative stress and mitochondrial dysfunction, and defective platelet aggregation in endothelial cells.^[64]^ Current evidence suggests that platelets release brain-derived neurotrophic factor (BDNF) upon activation. BDNF is a growth factor and a member of the neurotrophic factor family.^[65]^ After platelets activate BDNF, angiogenesis-related and inflammatory cytokines are released, while NR can regulate platelet activation, aggregation, secretion, and promote the expression of BDNF factor, exhibiting good antiplatelet activity and antidepressant effect.^[66,67^ ASD patients are associated with a higher prevalence of obesity, overweight, and cancer due to their endocrine disorders. Importantly, NR can regulate hormone levels through multiple pathways such as endocrine resistance and proteoglycans in cancer to increase gut microbiota diversity and regulate hormone imbalances and maternal metabolic disorders.^[68]^

There is growing evidence that NR has potential effects in the protection against ASD. Importantly, our study provides a preliminary theoretical basis and basis for new drug development and clinical trials for ASD and provides the foothold for future studies to explore the mechanism in treating underlying therapeutic effect of other Chinese herbal medicines and NR-rich vegetables and fruits. The diversity of ASD phenotypes and the complexity of molecular mechanisms emphasize the need for future studies to find reliable drugs and explore their mechanisms of action to improve the quality of life of families affected by ASD.

## 5. Conclusion

In summary, the present study found a series of novel targets and pathways for NR treating ASD and the mechanism of action on the human body suggests the involvement of multiple pathways and multiple targets. It was preliminarily predicted that NR could regulate the targets of CASP3, AKT1,ESR1,ACTB,MAPK3 and may modulate various pathways like estrogen signaling, thyroid hormone signaling pathway, prolactin signaling pathway, proteoglycan in cancer pathway, endocrine resistance pathway. Its mechanism of action are involving anti-oxidative stress response, anti-apoptosis, regulation of cell growth and metabolism, anti-inflammatory, balance hormone levels. At the same time, the molecular docking also corresponds to the predicted results, showing good target binding ability, which verifies the feasibility of the predicted components, core targets and their pathways in this study.

This study provides a potential biological basis for the further study of NR in the treatment of ASD, but still has some limitations. We used only modern bioinformatics methods to explore the effects of NR in the treatment of ASD by using network pharmacology and molecular docking. Firstly, data from online databases are based on reviewed and predicted data, and unconfirmed and unrecorded data were not included in our study. Secondly, although CASP3, AKT1, ESR1, ACTB, MAPK3, were identified as core targets, pharmacodynamic and molecular biology experiments need to be considered to further investigate our results. We believe that this topic has great research potential and application value.

## Author contributions

**Conceptualization**: Jialin Gai.

**Data curation**: Jinxiao Xing.

**Formal analysis**: Jialin Gai, Yangyang Wang.

**Funding acquisition**: Jiqin Tang.

**Methodology**: Jialin Gai, Junfang Lei, Chengdong Zhang.

**Software**: Jialin Gai, Junfang Lei.

**Supervision**: Jiqin Tang, Jinfei Zhang.

**Writing—original draft**: Jialin Gai.

**Writing—review & editing**: Jialin Gai, Jinxiao Xing, Yangyang Wang, Junfang Lei, Chengdong Zhang, Jinfei Zhang, Jiqin Tang.
